# Editorial: Cholangiocarcinoma a New Multimodality Approach

**DOI:** 10.3389/fonc.2022.935340

**Published:** 2022-06-22

**Authors:** Fabrizio Bronte, Sandro Sferrazza, Francesco Giuseppe Carbone

**Affiliations:** ^1^ Gastroenterology Unit, Ospedali Riuniti Villa Sofia –V. Cervello, Palermo, Italy; ^2^ Gastroenterology and Endoscopy Unit, Santa Chiara Hospital, Trento, Italy; ^3^ Unit of Surgical Pathology, Santa Chiara Hospital, Trento, Italy

**Keywords:** cholangiocarcinoma, radiology, oncology, endoscopy, multimodality, therapy, locoregional treatment

## Introduction

Cholangiocarcinoma (CCA) is one of the most aggressive cancers and is the second leading cause of primary liver cancer in the world. Several risk factors have been recognized, including primary sclerosing cholangitis (PSC), which is the most important risk factor in the western world, parasitic infections like *Opisthorchis viverrini* and *Clonorchis sinensis*, hepatolithiasis or Caroli disease, hepatotropic virus infection such as HBV and HCV, non-alcoholic fatty liver disease, and cirrhosis. The mechanisms by which these risk factors favor the development of CCA recognize ductal and periductal inflammation as the first factor that induces neoplastic transformation (1). In the last five years, the approach to CCA has moved toward radiological and histological diagnosis and treatment. CCA is often difficult to diagnose as it is asymptomatic in the early stages, meaning it is usually diagnosed in advanced stages and jaundice due to biliary obstruction is a common symptom. In this case, for example, jaundice requires specific treatment before starting chemotherapy, and endoscopists and interventional radiologists play a key role in these cases (2). Therefore, CCA requires a multimodality approach to precise diagnosis, staging, and treatment. Information about the site, dimension, stage, and histology are provided by radiologists and pathologists and are vital to be able to begin treatment.

This Research Topic focuses on the multidisciplinary management of cholangiocarcinoma, progress in diagnosis and treatment, and defines a new alliance for the CCA. We aimed to provide a platform for different authors by focusing on diagnostic and therapeutic aspects. Zhang et al. emphasize the urgent need for high-quality clinical trials and conversions from basic studies to clinical therapies for CCA through the analysis of literature using Machine Learning methods. Gao et al. examine patient derived xenograft (PDX) models. This new model provides a powerful platform for preclinical drug discovery and potentially facilitates the implementation of personalized medicine and improvement of survival for ICC cancer patients. We subsequently reviewed the literature with Ney et al., evaluating advances in the diagnostic pathway for suspected CCA as well as emerging diagnostic biomarkers for early detection, with a particular focus on non-invasive approaches. Xing et al. focused on the radiological aspects of cholangiocarcinoma, showing how a combination of DWI and hepatobiliary-stage enhanced imaging has higher sensitivity and accuracy to intrahepatic mass-forming cholangiocarcinoma diagnosis.

Li et al. review advances and highlight future directions for interventional treatment and Yang et al. discuss the safety of irreversible electroporation treatment in association with intraoperative biliary stent placement and how percutaneous biliary drainage (PTBD) tubes improve the quality of life of patients. Finally, in the context of the treatments proposed by Wu et al., Patient-Derived tumor Xenograft models (PDX) of cholangiocarcinoma represent new areas of basic research and individualized treatment of cholangiocarcinoma after surgery.

## Author Contributions

All authors listed have made a substantial, direct, and intellectual contribution to the work and approved it for publication.

## Conflict of Interest

The authors declare that the research was conducted in the absence of any commercial or financial relationships that could be construed as a potential conflict of interest.

## Publisher’s Note

All claims expressed in this article are solely those of the authors and do not necessarily represent those of their affiliated organizations, or those of the publisher, the editors and the reviewers. Any product that may be evaluated in this article, or claim that may be made by its manufacturer, is not guaranteed or endorsed by the publisher.
